# Parents’ Participation in the Sexuality Education of Their Children in Namibia: A Framework and an Educational Programme for Enhanced Action

**DOI:** 10.5539/gjhs.v8n4p172

**Published:** 2015-08-18

**Authors:** Linda Ndeshipandula Nghipondoka- Lukolo, Kimera Lukanga Charles

**Affiliations:** 1 Department of Family and Community Medicine, School of Medicine, University of Namibia, Windhoek, Namibia; 2 Department of Obstetric and Gynecology, School of Medicine, University, of Namibia, Windhoek, Namibia

**Keywords:** sexuality education, facilitation, communication skills, interpersonal relationship, knowledge, attitude, culture, behaviour, empowerment, self-awareness, self confidence, conceptual frame work, programme development

## Abstract

The purpose of the study was to empower rural parents to participate in the sexuality education of their children. The study was designed to be qualitative, explorative, descriptive and contextual in nature. It was performed in three phases. Phase 1 consisted of a situational analysis to explore and describe how parents provide sexuality education. Phase 2 consisted of the development of a conceptual framework that facilitated the development of an educational programme. In phase 3 the programme was implemented and evaluated, recommendations were made and conclusions drawn. The main findings revealed two themes: factors influencing parental participation in their children’s sexuality education, and the need for parental participation in their children’s sexuality education.

This article is part of series of three article stems from a study on the topic of sexuality education empowerment programme of rural parents in Namibia. The three articles have the following titles one: parent’s participation in sexuality education of their children: a situational analysis; two: parent’s participation in sexuality education of their children: a conceptual framework and an educational programme to enhance action, and three: parent’s participation in sexuality education of their children: programme implementation and evaluation.

The previous paper dealt with parent’s participation in sexuality education of their children: a situational analysis: the results from the in-depth interviews and focus group discussions on sexuality education with children and parents were presented. This paper focuses on describing Phase 2 and 3, namely the process of devising a conceptual framework for the development of an educational programme to empower parents to participate in the sexuality education of their children. Discussions included a description of the conceptual framework, based on the researcher’s paradigmatic assumptions, and the focus group and individual in-depth interviews results. The survey list suggested by Dickoff et al. (1968) consists of various elements which were employed in the conceptual framework, namely the context, agent, recipients, dynamics, procedure and a terminus. These elements were reflected in the “thinking map”.

## 1. Introduction

Conceptual framework in general is a real or conceptual structure intended to serve as a support or guide for the building of something that expands the structure into something useful, a framework is often a layered structure indicating what kind of programs can or should be built and how they would interrelate. A framework may be for a set of functions within a system and how they interrelate; the layers of an operating system; the layers of an application subsystem; how communication should be standardized at some level of a network; and so forth. The conceptual framework of the programme in this study was structured according to the survey list suggested by Dickoff, James and Wiedenbach (1968), which facilitates the identification and categorisation of major concepts for further refinement, thus ensuring logical development of the sexuality educational programme

## 2. Classifications and Definitions of Central and Associated Concepts

The identification of the central concept was based on the empirical data of the first phase. After analysis it became clear that parents and children feel that parental participation and involvement in sexuality education is inadequate, that the interpersonal relationships between parents and their children are poor, that parental attitudes and behaviour concerning sexuality are negative, that parents lack knowledge about sexuality-related issues and that cultural beliefs can negatively affect parents’ participation in their children’s sexuality education. The above factors resulted in a lack of confidence, fear, insecurity and uncertainty amongst parents. Furthermore, these factors are known to create resistance to any change, as parents who are to change do not know what to do, how to do it, where to start, what the effect will be and how their children and they themselves will benefit from the change to be introduced.

However, this study has found that change is necessary and a great need for parents to participate in sexuality education of their children were identified. This need can be met through providing proper information, motivation and encouragement, promotion of constructive interpersonal relationships and the discouragement of negative behaviour that may jeopardize the health status of the children such as cultural beliefs. Participation and involvement enhance the feeling of ownership that may allow parents to be responsible for the health of their children and the nation of tomorrow. The experiences reported by parents during this study indicate that parents need to be empowered in order to be able to participate in the sexuality education of their children.

However, empowerment starts with the self (Swanepoel, 1992). Thus the parents (recipient) must first possess the necessary traits (SIECUS, 1999). SIECUS (1999) identifies the following characteristics to be required to enable parents to be both responsive and responsible for constructing the necessary knowledge and skills: responsibility, flexibility, mutual trust and respect, understanding, commitment, strength, ownership, motivation, openness, steadiness, confidence, willingness, constructive interpersonal relationships and communication, patience, supportiveness, safety and security and role modeling. Through an interactive process, children will gain new insights into knowledge of and skills in sexuality-related issues. Children will become independent and autonomous in managing their own health (Wertsch, 1995; Blekinsop, 2004; Cummins et al., 2003).

The concept of empowerment has different meanings in different contexts. For the purposes of this study, empowerment refers to assisting parents to provide sexuality education to their children; making parents capable of rendering sexuality education.

## 3. The Researcher’s Reasoning Map

The survey list of Dickoff et al (1968), which includes the ingredients of context, agent, recipient, dynamic, procedure of the activity and terminus, was used as a basis for the formulation of the conceptual framework. The aim of this framework was to identify and categorise major and related concepts for further clarification of and reflection on the programme. The researcher’s reasoning map represents the interaction between the agent and recipients, which is contextualised within a specific framework and procedure. The context determines the procedure to be followed whilst acknowledging the dynamics underpinning the interaction and facilitation process, with the intention of attaining a specific outcome or to reach a specific goal (Fucci, 2000).

**Figure 1:**
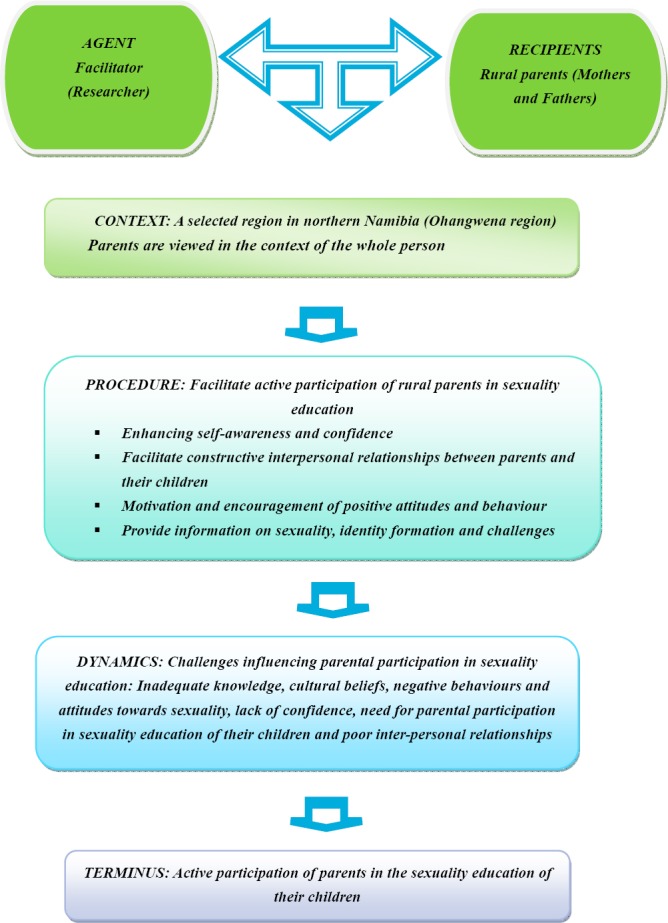
Researcher’s reasoning map

The researcher’s thinking map as developed for this study is outlined in figure 1. The survey list of Dickoff et al (1968) was adopted as a basis for the formulation of the researcher’s thinking map. Dickoff indicates the six survey lists as follows:

### 3.1 Agent: Researcher

**Figure 2:**
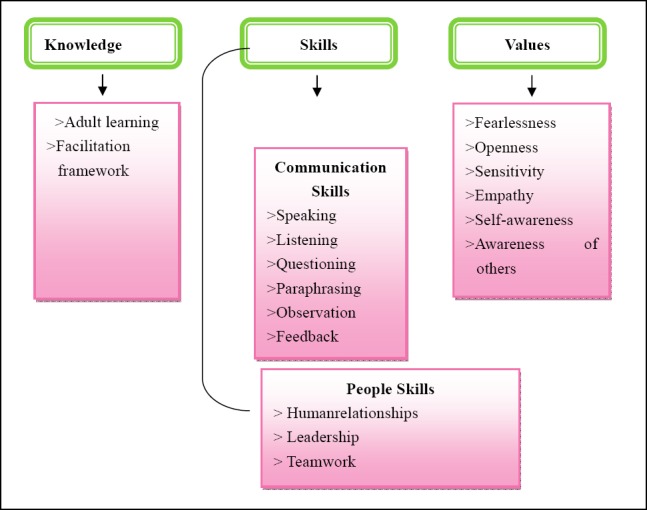
Characteristics of an agent

The term “agent” refers to the person who performs the activity (Dickoff et al., 1968). For the purpose of this study, the agent is the researcher, who has to provide the activity, namely an educational programme for empowering parents to participate in the sexuality education of their children in the Ohangwena region. This requires, firstly, that she possess the personal qualities needed to build constructive interpersonal relationships with the recipients, namely the parents. Each of these attributes is displayed in Figure 2 illustrate the Characteristics of an agent:

**Knowledgeble**

“Knowledge” is the theoretical framework, the “what to do” and the “why”. Knowledge is generated through the participants’ experiences rather than being presented as a product by an expert, the facilitator, to the participants (Rooth, 1995).

The facilitator is the resource who promotes the active learning process in which the participants are engaged by providing the structured framework for them to develop, explore, experiment and learn (Meyer, 2004, p. 35).

In order for the agent to provide guidance and support to parents, she should be able to incorporate her knowledge in guiding and supporting parents. In providing support and guidance, the facilitator should have certain personal qualities that enable her to carry her role effectively. One of the personal qualities that the agent should have is the ability to build constructive interpersonal relationships, should be able to establish and maintain positive relationships based on mutual trust, to work collaboratively, to accept compromises when appropriate, to communicate effectively and to listen to problems encountered by recipients (parents) (Taris, 2000; Ritchie, 2006; Chin & Kramer, 1995).

In addition to the knowledge and personal qualities that an agent should have, she also requires a variety of characteristics in order to be able to facilitate the empowerment of parental participation and involvement in sexuality education. These characteristics are: Open-mindedness, knowledge, trust and respect for relationships, objectivity, courage, commitment and confidence, communication skills such as speaking, listening, questioning, paraphrasing, observing, feedback and summarising.

People skills: An effective facilitator must listen well and have a genuine interest for how others think and feel. The facilitator for this programme has a pleasant personality and enjoys interacting with other people. This helped the facilitator to be able to lead the group to achieve its objectives and to show the group direction and inspire them to work together (Byrne, 2007).

Attitudes of facilitator: “Attitude” refers to people’s positive or negative evaluation of any aspect of the physical or social world shaped by their experience and interpersonal relationships, and is stored as memory (Harrison, 2004; Whitaker et al., 1999).

Empathy: Involves a deep understanding of the feelings and thoughts that make up the inner experiences of the participants, recognising these as experiences that the facilitator herself may have had (Henry, 1998; Atherton, 2002). This means that the facilitator senses and accepts the experiences of the participants without becoming too emotionally involved. An effective facilitator is also guided by the value of Fairness: All participants should be treated in a fair and consistent manner, and preference should not be shown to any individual or group (Meyer, 2004).

### 3.2 Recipient: Rural Parents in the Ohangwena Region

Meyer, (2004) defines the term “recipient” as a beneficiary of something, an addressee, and receiver. In this study rural parents of children between the ages of 12 to 16 in the Ohangwena region are the recipients of this programme. Parents should possess certain characteristics (Bradley, 2005) in order to benefit from the programme. The following characteristics are required to enable the recipients to be both responsive and responsible for constructing the knowledge and skills needed to be able to participate and be involved in the sexuality education of their children:

Responsibility: Parents are responsible for the education of their children, thus they should participate in the provision of sexuality education for their youth.

Motivation: This characteristic is a combination of desires, values, and beliefs that drives parents to do their work (Wood, 2002). Parents need to be motivated, in order to take part in sexuality education of their children (Meyers & Thomas, 1998).

Understanding: Parents need to understand their children’s uniqueness and totality as a human being (Meyer, 2004). Every human being has got his/her own need and problem that is the reason why parents need to be aware of the uniqueness of their children and to attend to their needs differently.

Openness: This characteristic includes: freedom of expressing oneself, freedom to discuss and to talk openly with ones child, and being open to ideas from all people no matter how wild or unrealistic these ideas may be seen. Parents must also be accessible so that everyone - no matter what their views - will feel that they can approach them with almost any issue, no matter how serious or sensitive (Meyer, 2004).

Confidence: This characteristic is a combination of believing and trusting.

Willingness: Parents need to exercise willingness to participate in sexuality education of their children.

Mutual trust and respect: Parents must epitomise the value of respect, should build on the principle of respect for other people (Meyer, 2004). Parents should respect themselves and their children.

Effective interpersonal relationships and communication: Good interpersonal relationships provide the basis for effective and open communication between parents and their children (Rae, 1997).

### 3.3 Context: Ohangwena Region

Context is a situation, a framework, a milieu, an environment or a background. For the purpose of this study, the context is defined as an environment or a milieu consisting of parents who have children between the ages of 12 and 16 years living their homes in the Ohangwena region, where the programme is to be implemented. This study is contextual in nature. Data were obtained from both fathers and mothers between the ages of 29 and 59, and children between the ages of 12 and 16, living in the Ohangwena region. The Ohangwena region was selected because the rural areas are most disadvantaged when it comes to the accessibility of health services. Most of the people living in rural areas do not have access to health services, and most of the Namibian youth reside in northern Namibia.

### 3.4 Procedures

For this study, procedures were needed to facilitate active participation of rural parents in the sexuality education of their children. These procedures were identified from the data analysis as the results of the situational analysis namely, to enhance self-awareness and confidence of rural parents, to facilitate constructive interpersonal relationships between parents and their children, to motivate and encourage positive attitudes and behaviours, and to provide information on sexuality. The programme was structured around the experiential approach to learning as suggested by Kolb. Thus, the programme was based on experiential learning and was conducted in the form of a workshop. (Kolb’s experiential learning cycle is discussed in Chapter 5). The workshop, which incorporated an orientation phase, a working phase and termination phase, was implemented over three days, and was structured as a helping relationship. The workshop was organised around a particular base, and incorporated a structured and sequenced set of activities and exercises designed to equip parents with knowledge of sexuality and skills to provide sexuality education. The workshop was carefully planned to meet specific goals, which were based on the needs of the participants.

#### 3.4 1 Enhancing Self-Awareness

Enhancing self-awareness is crucial to psychological insight and self-understanding (Gouws, 2000), because enhancing self-awareness motivates people to reduce the discrepancy between how they actually behave and how they desire to behave (Moskowitz, 2005). It is crucial for enabling parents to know who they are and why they exist, and helping them to be aware of their responsibility regarding the sexuality education of their children. Dolinsky, 2000) states that before a person can lead or help other people, he/she has to discover himself/herself.

#### 3.4.2 Facilitating Constructive Interpersonal Relationships

Interpersonal relationships can be defined as a relatively long term association between two or more people. This association may be based on emotions like love and liking, regular interactions, or some other type of social commitment. Constructive interpersonal relationships are the driving force behind parental participation and involvement in the sexuality education of their children (Moskowitz, 2005). A relationship is normally viewed as a connection between two individuals, such as parent and their children (Fisher, 1995; Forsyth, 2006). These relationships usually involve some level of interdependence. People in relationships tend to influence each other, and share their thoughts, feelings and engage in activities together. People may be interdependent if they are sharing mutual and common goals. In this study constructive interpersonal relationship are the driving force behind parental participation and involvement in the sexuality education of their children.

#### 3.4.3 Motivation and Encouragement of Positive Attitudes and Behaviour

Firstly, people must be aware of the importance of attitude. It is important to remember that attitude is everything, whether positive or negative shows in our daily lives. People cannot change the past or how others act, but they can change their reactions and attitudes towards the past or experiences. A positive attitude is a vital asset at any time. People should portray a positive attitude when they present themselves (Yowell, 2004).

Secondly, parents must take responsibility for their attitude as well as their actions. This will give strength and control during time of change. When change occurs, people may experience feelings of fear, anger, sadness, or resistance, as well as relief, hope or excitement (Yarcheski & Mahon, 2009). Parents need a positive attitude, which can help them to shape the future of their children, instead of being held back by allowing negative feelings to affect their attitude and behaviour. They must be positive role models for their children and others, as the future and hope now lies in the hands of children.

The results of this study clearly showed that parents need to change their behaviour and attitudes towards sexuality-related issues. They should talk to their children, because this will improve the sexuality health of children. However, talking openly about sexuality has not always been easy. Meanwhile, Rew (2005) and Taravera (2002) are of the opinion that changing ways of discussing sexuality-related issues does not mean that culture and tradition will be threatened. Individuals’ commitment to behaviour change is reinforced by being involved in planning, by doing self-monitoring and by conducting frequent “self-awareness” checks (Quinn, 2000; Schoeberlein, 2004). They further stated that positive reinforcement can help change behaviour, and that it requires an environment that supports activities and the internal motivation to live a long life (Greathead, 2002).

#### 3.4.4 Provision of Information About Sexuality

Parents need to gain knowledge and skills on aspects related to sexuality education. Such knowledge and skills will reduce feelings of fear, incompetency, inadequacy and embarrassment and will enhance constructive interpersonal relationships between parents and their children. Parents will develop a sense of self-confidence, ownership, self-determination and self-control and positive attitudes towards sexuality. Parents need information on child development and the challenges that may occur during adolescence. Such as:

•Identity formation

Identity formation is the process of developing a distinct personality as an individual regarded as a persisting entity (known as personal continuity) in a particular stage of life in which individual characteristics are attained by which a person is recognised or known (such as the establishment of a reputation). This information will help parents to know that children are unique; that personalities differ from one individual child to the next. This process defines individuals to others and themselves (Erikson, 2007; Goossens, 2008). Parents will be given information on developmental stages, namely, physical, social, emotional and interpersonal identity development. This helped parents to address the different needs of their children.

•Challenges during adolescence

Adolescence is full of challenges for any child. The change is fast, everywhere, and hard to keep up with. The body changes in response to increasing hormone levels; thinking processes change as the child is able to think more broadly and in an abstract way; social life changes as new people and peers come into scope. Yet the child needs to deal with every single one of these changes, all at the same time. With their willingness to help, that’s where the parents come in, who have “been there”, with the life experience, maturity and resources (Ozbayrak, 2006).

Parents will be equiped with knowledge on teenage pregnancies, HIV and AIDS; sexuality transmitted diseases, alcohol and drug abuse, sexuality harassment and discipline. Parents will be given information to enable them to combat those challenges and to be able to deal with them when they occur (Dickson et al, 2002; Steinberg, 2008).

### 3.5 Dynamics

“Dynamics” refers to the energy source or motivation for the activity (Dickoff et al., 1968). The dynamics that emerged from this study are the opposite of the themes discussed in the first article of the study, namely: Challenges influencing parental participation in the sexuality education of their children, focused on: inadequate knowledge on sexuality education, cultural beliefs of parents, negative attitudes and behaviour concerning sexuality, poor interpersonal relationships between parents and their children.

#### 3.5.1 Inadequate Knowledge on Sexuality Issues

“Knowledge” refers to a body of facts or ideas acquired through study, investigation, observation or experience. Parents need knowledge of sexuality-related issues. If they do not have adequate knowledge about sexuality, it is difficult for them to talk to their children. This study confirms this point, because it found that many parents do not participate in the sexuality education of their children because they do not know enough, they do not know how to explain what they do know, and they feel incompetent and embarrassed. Bandura (1992) is of the opinion that initiating conversations about the facts of life may be difficult for some parents because they did not grow up in an environment where the subject was discussed. As the challenges faced by today’s adolescents have increased, parents must talk to their children in order to prevent and control the dangers that Namibian children are exposed to. HIV and AIDS, Sexual transmitted infections, teenage pregnancies, sexual harassment and more can only been overcome if parents participate in the sexuality education of their children (Erickson, 2007; Wight, 2003; Fitzsimons, 1991).

#### 3.5.2 Cultural Beliefs

“Culture” refers to attitudes, beliefs, values and behaviours that are characteristic of a particular social group. Culture is transmitted from generation to generation and shapes and influences perceptions and behaviours. This study revealed that culture is an obstacle to sexuality education. Most of the parents who participated in this study do not provide sexuality education, because it is culturally a taboo for parents to talk to their children about sexuality-related issues (Wight et al., 2003; Fitzsimons, 1991). The researcher believed that if parents have proper information on sexuality and increase their self-awareness, that change may occur and parents may begin to participate in the sexuality education of their children.

#### 3.5.3 Negative Attitudes and Behaviours Towards Sexuality

Attitudes can be defined as a person’s mood and feelings towards things, circumstances or people. A positive attitude is a priceless possession. During this time of societal change, it is sometimes easy to allow circumstances to rob us of this possession. Parents need to change their negative attitudes and behaviours towards sexuality in order to be able to talk to their children (Forrest, 2006; Jemmott & Jemmott, 2002; Kirby et al., 2007; Kirby et al., 1991).

#### 3.5.4 The Need for Parental Participation in Sexuality Education

The goal of the facilitator was to equip parents with the skills and knowledge on sexuality-related issues necessary to overcome factors influencing parental participation in sexuality education, to encourage positive attitudes and behaviour towards sexuality, to enhance self awareness, to motivate interpersonal relationships and open communication between parents and their children and to build up their confidence (Exeter, 2001; Holtzman & Robinson, 1991). They will be discussed as follow:

**Figure 3:**
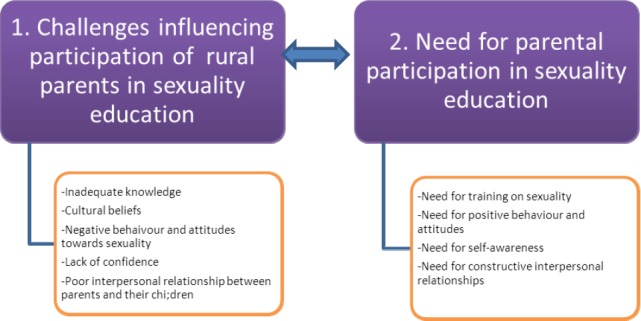
Dynamics

Interaction is a dynamic process, which entail communication, leadership, reasoning and logical argumentation as well as competence and effectiveness. The researcher should facilitate these qualities, as this will enable parents to see the importance of participation in sexuality education. Addressing the dynamics of challenges that influencing parental participation in sexuality education of their children en able parents to have proper information on sexuality. Parents became aware of the importance and the advantages of participating in sexuality education of their children. Interpersonal relationship enhances effective communication, active participation enhances confidence, continuous interaction and feeling of ownership.

### 3.6 Terminus

“Terminus” refers to the last stop, the end or the finishing point (Dickoff et al., 1968). The terminus of this programme is active participation of parents in the sexuality education of their children. If parents actively participate in providing sexuality education to their children, children will act responsibly in their sexuality activities. They will be able to protect themselves against Sexuality Transmitted Diseases, unwanted pregnancies and HIV and AIDS and children who have discussed sexuality-related issues with their parents are less likely to participate in risky sexuality behaviour and may delay sexuality activity.

## 4. Relationship Between Concepts

**Figure 4:**
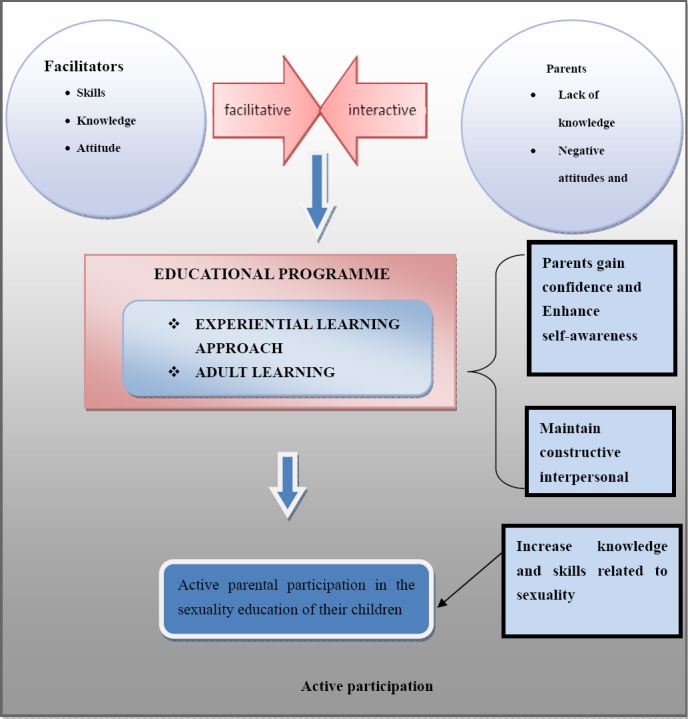
Graphic representation sexuality educational programme development process related as well as the relationships between concepts

The relationship between the concepts is graphically represented in Figure 4 and discussed under the haracteristics of an agent. It is clear from the results that parents are not adequately participating in the sexuality education of their children. This is due to fear, lack of knowledge, cultural beliefs, negative attitudes towards sexuality and poor interpersonal relationships between parents and their children. They also expressed the need to acquire the necessary skills to be able to participate in the sexuality education of their children.

“Active participation” refers to taking part, being actively involved, or sharing with others. The end result of this study will be the active participation of parents in the sexuality education of their children. If parents actively participate in providing sexuality education to their children, children will act responsibly in their sexuality activities. They will be able to protect themselves against Sexuality transmitted diseases, unwanted pregnancies and HIV and AIDS. Authors like Braithwaite and Baxter (2006, p. 56) and Harrison (1998, p. 86); Gagnon, (1997); Hickman-Brown, (2001) are of the opinion that children who have discussed sexuality-related issues with their parents are less likely to participate in risky sexuality behaviour and may delay sexuality activity.

**Concluding statement on conceptual phrame work development**

The ability to communicate is not unique to humankind, but we have a sophistication that far surpasses all other species. It enables us to move beyond events taking place at the time. Parents can share ideas, knowledge, beliefs and other opinions about happenings in the distance past and possibility for future; about events here or in some other places; about the particular or general; the concrete or the abstract. It also enables us to make meaningful contact with others through establishing, maintaining relationships (Owen, 1994:35). It will ensure continuity and constructive relationship between parents and their children.

Parental empowerment in participation and involvement in sex education may be resulting in competent parents, skillful parents, possess knowledge and proper information. Interpersonal relationship between parents and their adolescents enhances open communication, they will be able to discuss their problems and share experience openly. This may result in a responsible child.

## 5. Sexuality Educational Programme Development

This part of the article focuses on the development of an educational programme to empower rural parents to participate in the sexuality education of their children. After analysing the data and formulating a conceptual framework, it was possible to develop a programme based on the themes identified. The programme development was based on the survey guide suggested by Dickoff et al (1968, p. 423). The development of the educational programme serves as an intervention to empower parents to participate in the sexuality education of their children. The desired outcome of the programme is to ensure that parents actively participate in the sexuality education of their children. The required qualities and skills of the facilitator, the nature of experiential learning as well as the specific characteristics and needs of adult learning had to be accommodated during the development of the programme.

### 5.1 Purpose of the Educational Programme

The purpose of this programme is to empower rural parents to participate in the sexuality education of their children. After meticulous evaluation of the data collected in Phase 1 of this study, four areas to be addressed in the programme were identified. The areas included activities, such as facilitating constructive interpersonal relationships between parents and their children, enhancing parents’ self-awareness and confidence regarding sexuality education, identity formation and challenges faced by adolescents in Ohangwena region in Namibia. Swanepoel (1992, p. 2) is of the opinion that a programme is always developed with concrete purposes in mind, and that the researcher also subconsciously arrives at “abstract goals”, which refer to the stable and permanent outcome of the programme. The long term goal of this programme is active parental participation in the sexuality education of their children (Fawcett, 1995).

### 5.2 Context for the Implementation of the Programme

The “context” refers to the frame of reference in which the activity takes place. The context of the implementation of this programme is the Ohangwena region. However, facilitation will take place within institutional settings in the region, such as health centers, churches or schools. The facilitator possesses the values, skills and knowledge necessary to educate rural parents about sexuality-related issues.

### 5.3 Description of the Educational Programme Process

**Figure 5:**
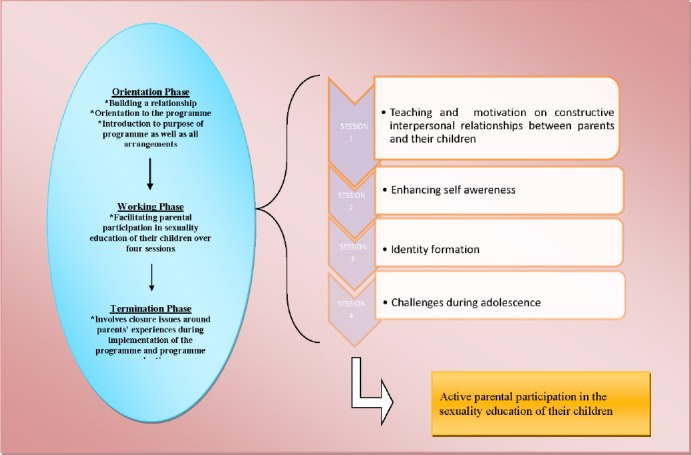
Graphic representation of an educational programme development process

The educational programme involves a three-phase process, consisting of an orientation phase, a working phase and a terminal phase. See Figure 5. This is a cyclical process typical of each workshop contained in the broader implementation of the programme. The programme consists of three day workshops, which are also contained in a framework of an orientation phase, a working and a termination phase.

#### 5.3.1 Orientation Phase

In the orientation phase the facilitator and rural parents met in a selected venue in the Ohangwena region to be able to attend an introductory meeting where aspects related to the purpose, goals and logistical arrangements of the implementation of the programme were discussed. The meeting was held in the morning on the day before the commencement of the workshop. Participants were informed about the purpose of the programme and were asked to consent to participation in the development of the programme. The logistical arrangements, including broad outline and expectations of the programme, were reviewed. The ground rules and expectations were discussed and repeated at the beginning of each session. The facilitator used ice breaker activities in each of the sessions in order to afford participants the opportunity to settle down and get comfortable with each other.

#### 5.3.2 Working Phase

The programme is based on the experiential learning approach within the paradigm of adult learning, and structured as a three day workshop. The programme addresses the needs identified from an analysis of Phase 1 of the study. The working phase is centered on teaching rural parents skills to enable them to provide sexuality education. Parents acquired skills through active participation in group discussions and presentations, as suggested in the experiential learning cycle. The programme addresses the following aspects:

- **Session 1** focused on an introduction and orientation of the programme content, the establishment and maintenance of healthy interpersonal relationships and communication skills.
- **Session 2** dealt with activities to enhance self- awareness, motivation and encouragement of parents to participate in their children’s sexuality education.
- **Session 3** emphasised identity formation, which helped parents to gain knowledge of the different stages of development during childhood.
- **Session 4** deliberated on challenges faced during adolescence, evaluation of the programme and closure of the workshop.

The facilitation was done within the framework of the experiential learning approach, in acknowledgement of the fact that ensuring freedom within the pre-planned structure of the programme is important (Hobbs, 1992, pp. 93-94; Lamberski, 2002, p. 23).

#### 5.3.3 Termination Phase

During the termination phase, all the issues around parents’ experiences during the implementation of the programme were summarised and reflected upon. As part of reflection process, the programme was evaluated. This phase was conducted in line with the nature of the experiential learning process, in which group input helps the facilitator to stay on track and allows participants to come to terms with the process (Hobbs, 1992, p. 101; Meyer, 2004, p.30). Rae (1997, pp. 7-9) states that evaluation provides first-hand information about how participants experience what you as facilitator and/or programme developer have to offer.

## 6. Educational Approach

The educational programme took cognizance of the experiential approach to learning within the paradigm of adult learning, as suggested by Smith, 2002, p. 28). Experiential learning refers to learning opportunities where participants learn from and through personal and group experience and by reflecting on what has been learned (Rooth, 1995, p. 3; Smith, 2002, p. 22). The focus in experiential learning is on building on existing strengths and on the life experiences of the participants.

### 6.1 Experiential Learning Approach

Experiential learning is a learning theory and philosophy, which facilitates learning from direct experience through active participation in the learning process, and by reflecting on what has been learnt in the context of group work and sharing ideas on the learning process (Hobbs, 1992; Kirby, 2000; Joplin, 1981; Gass, 2002). Kolb’s learning theory sets out four distinct learning styles (or preferences), which are based on a four-stage learning cycle (which might also be interpreted as a “training cycle”. Kolb and Kolb (2001) and Kolb, (1984) proposed that the four interdependent series of steps essential for effective learning include concrete experience, reflective observation, abstract conceptualisation and active participation.

In this respect, Kolb’s model of experiential learning is particularly elegant, since it offers both a way to understand individuals’ different learning styles, and also an explanation of a cycle of experiential learning that applies to us all. Kolb includes this “cycle of learning” as a central principle of his experiential learning theory, typically expressed as a four-stage cycle of learning, in which “immediate or concrete experiences” provide a basis for “observations and reflections”. These “observations and reflections” are assimilated and distilled into “abstract concepts”, producing new implications for action which can be “actively tested”, in turn creating new experiences (Wight et al., 2003; Kolb & Kolb, 2001; Lamberski, 2005; Kelly, 1997).

**Figure 6:**
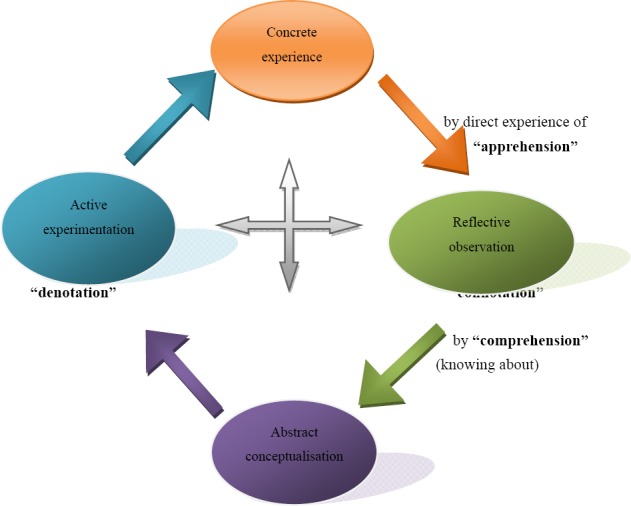
Kolb’s (2006) experiential learning cycle

Kolb and Kolb (2001) says that ideally (and by inference) this process represents a learning cycle or spiral where the learner “touches all the bases”, for example, a cycle of experiencing, reflecting, thinking, and acting. Immediate or concrete experiences lead to observations and reflections. These reflections are then assimilated (absorbed and translated) into abstract concepts with implications for action, which the person can actively test and experiment with, which in turn enable the creation of new experiences (Hobbs, 1992, p. 56; Smith, 2002, p. 32).

The approach was implemented by the facilitator, who maintained a favourable and conducive environment, provided the structure, recourses, and activities and asked appropriate questions that allowed parents to reflect on their experiences and to make sense of their personal learning. The components of the experiential cycle will now be discussed in detail.

#### 6.1.1 Concrete Experience

The process of experiential learning uses the existing knowledge (concrete experience) and competencies of group members. It refers to persons’ planned or unplanned actual experiences. Concrete experience corresponds with “knowledge by acquaintance”, direct experience (or “apprehension” in Kolb’s terms), as opposed to “knowledge about something”, which is theoretical, but perhaps more comprehensive (hence “comprehension”) and represented by abstract conceptualization (Hobbs, 1992, pp. 1-2; Willis & Ricketts, 2004).

This type of learning acknowledges and welcomes values and uses the existing knowledge and competence of every parent in the group. The value of experiential learning lies in the fact that the participants place more belief in the knowledge they have discovered for themselves than in the knowledge presented by experts (Rooth, 1999, p. 75; George, 1990).

This approach suggests that rural parents have concrete experiences. During the workshop parents generated knowledge through other participants’ experiences. They learned from one another’s experiences so that all learning processes became a shared learning experience for the whole group. The parents shared experiences of different aspects relating to sexuality with the group, and so came to understand what sexuality education entails.

#### 6.1.2 Abstract Conceptualization

Abstract conceptualization emerges out of reflection and can be tested actively in the fourth stage. Abstract conceptualization “is the effort to make sense of experiences by trying to theorize, to generalize, to look forwards for and construct alternatives” (Smith, 2004; Greenway, 2006b). Rural parents were able to gain insight as they were given the freedom to express how they provide sexuality education and made suggestions for possible changes.

#### 6.1.3 Reflective Observation

Through reflection, parents were empowered to plan, manage and evaluate their own learning and actively assimilate external knowledge into their own internal frame of references, thus learning to accept full responsibility for their own learning. Reflection was valuable in this programme because in an atmosphere of reciprocal sharing parents learnt to explore and express their feelings, focused on what they had learnt and what insights they had gained. Reflection enabled rural parents to make sense of past experiences and developed them for the future by making it possible for them to consider changing inappropriate behaviour. Parents consolidated and internalised the learning experience, thus ensuring lasting and meaningful learning (Rooth, 1999, p. 98; Dolinsky, 2000, p. 73). Reflective learning is based on the assumption that learning as an activity involves the whole person as a thinking, feeling, active being (Whitaker et al., 1999, p. 107).

#### 6.1.4 Active Experimentation

Active experimentation transforms the theory of abstract conceptualization by testing it in practice and relates to its denotations. The abstract conceptual analysis is tested actively. Active experimentation includes doing things in the real world, deciding what works and what does not, making plans and implementing those plans (Kolb & Kolb, 2001). Participants reflected on their own experience by demonstrating on how they communicate with their children. By demonstrating this within the group, members reflected on how they might manage better by using the programme strategies, which include communication skills. Kirby et al. (1994, p. 129) are of the opinion that consistent exposure to activities related to life experience clarifies and personalises these concepts so that they become part of the (personal) knowledge base. Rural parents increase their understanding of the self and their external world (Hobbs, 1992, p. 3).

This may generate enduring provides a model and a language through which participants reflect on the experience and participate in the learning process together in an environment of mutual support (Hobbs, p. 2-3). It is the researcher’s hope that the knowledge about sexuality which rural parents gained from this programme will lead to changes in their lives.

### 6.2 Parents as Adult Learners

According to Knowles (in Atherton, 2002, p. 12) two key differences in the ways that adults and children approach learning are that adults desire to be self-directed and want to take responsibility for decisions. Courses for adult learners are sensitive to these desires and designed to permit some autonomy in how participants approach and schedule their learning activities. Knowles’ lists the following assumptions on adult learning: adults need to know why they need to learn something, adults need to learn experientially, adults approach learning as problem-solving and adults learn best when (they believe that) the topic is of immediate value.

According to Meyer (2004, p. 35) adults learn better in the following conditions:

- Adults need to be respected and treated as people who have knowledge and experience.
- Adults need information that is useful to them, in other words, information they can use in their personal lives or at work.
- Adults need to be allowed to relate their own experiences and situations.
- Adults need to be allowed to listen to suggestions and decide what is useful to them.
- Adults need to contribute to the learning process, i.e. to give input and ideas to add value to the learning process.
- Adults need to cover information that will help them achieve their future objectives, such as career goals or other personal life challenges.

The facilitator of this programme was sensitive to the needs and styles of adults in order to get maximum results on the objectives. The facilitator made use of teaching strategies, such as role play (autonomy, experience) group work, feedback and self-evaluation to ensure active participation and effectiveness of the programme.

## 7. Conclusion

In this paper the researcher described the development of conceptual framework and an educational programme to empower parents in the Ohangwena region to participate in the sexuality education of their children. The discussion included a description of the conceptual framework based on the researcher’s paradigmatic assumptions and the focus group and individual in-depth interviews. The survey list suggested by Dickoff et al (1968, p. 435) encompasses various elements that were employed in the conceptual framework, namely the context, agent, recipients, dynamics, procedure and terminus. These elements were reflected in the “thinking map”.

The researcher defined and described the educational programme and concluded with a discussion of the experiential learning approach. The learning characteristics of adult learners were described in detail, so as to ensure that the facilitator is aware and sensitive to the requirements, as set out in the development of the conceptual framework of the programme. The next paper deals with the implementation and evaluation of an educational programme.
